# Ocular manifestations of vitiligo: a systematic review

**DOI:** 10.1186/s12886-023-02777-9

**Published:** 2023-03-27

**Authors:** Tessa LeWitt, Robert Tauscher, Gracious Obiofuma, Jonna Peterson, Ramez Haddadin, Roopal V. Kundu

**Affiliations:** 1 Department of Dermatology, Northwestern University Feinberg School of Medicine, Chicago, IL USA; 2 Department of Ophthalmology, Northwestern University Feinberg School of Medicine, Chicago, IL USA; 3 Galter Health Sciences Library and Learning Center, Northwestern University Feinberg School of Medicine, IL Chicago, USA

**Keywords:** vitiligo, dry eye disease, melanocyte, glaucoma, cataract

## Abstract

Vitiligo is a disorder characterized by loss of epidermal melanocytes, resulting in depigmented macules and patches. While the relationship between ocular pathology and vitiligo has been demonstrated in conditions such as Vogt-Koyanagi-Harada and Alezzandrini syndromes, the ocular associations of non-syndromic vitiligo are incompletely understood. We conducted a systematic review to comprehensively describe the structural and functional changes seen in the eyes of patients with vitiligo, to identify patients at heightened risk for ocular disease, and to provide an approach to management of ocular manifestations of vitiligo. Overall, the strongest link between vitiligo and ocular pathology seems to lie with dry eye disease and pigmentary abnormalities of various ocular structures, especially the retinal pigment epithelium. Normal-tension glaucoma may also be more prevalent in the vitiligo population. The available literature did not provide conclusive evidence for increased risk of cataracts or uveitis. Aside from the impact of symptomatic dry eye disease, it seems unlikely that there are significant functional consequences of these ocular manifestations such as impaired visual acuity or visual fields.

## Background

Vitiligo is an acquired, autoimmune and chronic disorder that is characterized by skin and hair depigmentation secondary to epidermal melanocyte destruction. Importantly, melanocytes – neural-crest-derived, melanin-producing cells – are found in a variety of anatomic locations other than the skin, including mucosa, cardiac valves [1, 2], the inner ear [3], and the uveal tract of the eye [4]. These ocular melanocytes are responsible for eye pigmentation and function to protect against ultraviolet radiation and oxidative damage [4]. Their presence in the uveal tract suggests that any process involving the destruction of melanocytes, such as vitiligo, may have ocular effects. Indeed, the relationship between ocular pathology and vitiligo is demonstrated in conditions such as Vogt-Koyanagi-Harada and Alezzandrini syndromes, which feature elements of ocular inflammation and vitiliginous skin. This relationship, though, may not be limited to specific syndromes.

The aim of this systematic review (SR) is to comprehensively describe the structural and functional changes identified in the eyes of patients with vitiligo and to provide an approach to management of ocular manifestations in vitiligo.

### Main text

This review was registered with Prospero (CRD42021233327). A medical librarian (J.P.), with training in SR methodology, searched MEDLINE, EMBASE, and the Cochrane Library for articles published up until February 5, 2021. The following subject terms and keywords were used: vitiligo, eye, ocular, and vision. During review, eligible studies included patients with vitiligo and ocular manifestation(s). Case reports, case series, and reviews were excluded. Randomized controlled trials, prospective analyses, retrospective analyses, case control, cohort, cross-sectional, and non-controlled before-and-after studies published in peer-reviewed journals in the English language were included. Gray literature was also included.

A pair of independent reviewers (T.L., G.O.) independently screened all abstracts. When primary reviewers could not reach a consensus, a third independent reviewer (R.K.) adjudicated the results. For abstracts identified as potentially relevant, two authors (T.L., G.O.) independently reviewed full-text articles, determining final inclusion by consensus. When consensus could not be reached, a third independent reviewer (R.K.) adjudicated the results. Two authors (T.L., G.O.) extracted study characteristics (authors, year, country, study design, sample size, patient characteristics, methods, intervention, primary endpoints, and results) from the final list of included studies. The authors independently abstracted the data and then reviewed and confirmed the accuracy of the others’ work.

**Fig. 1:**
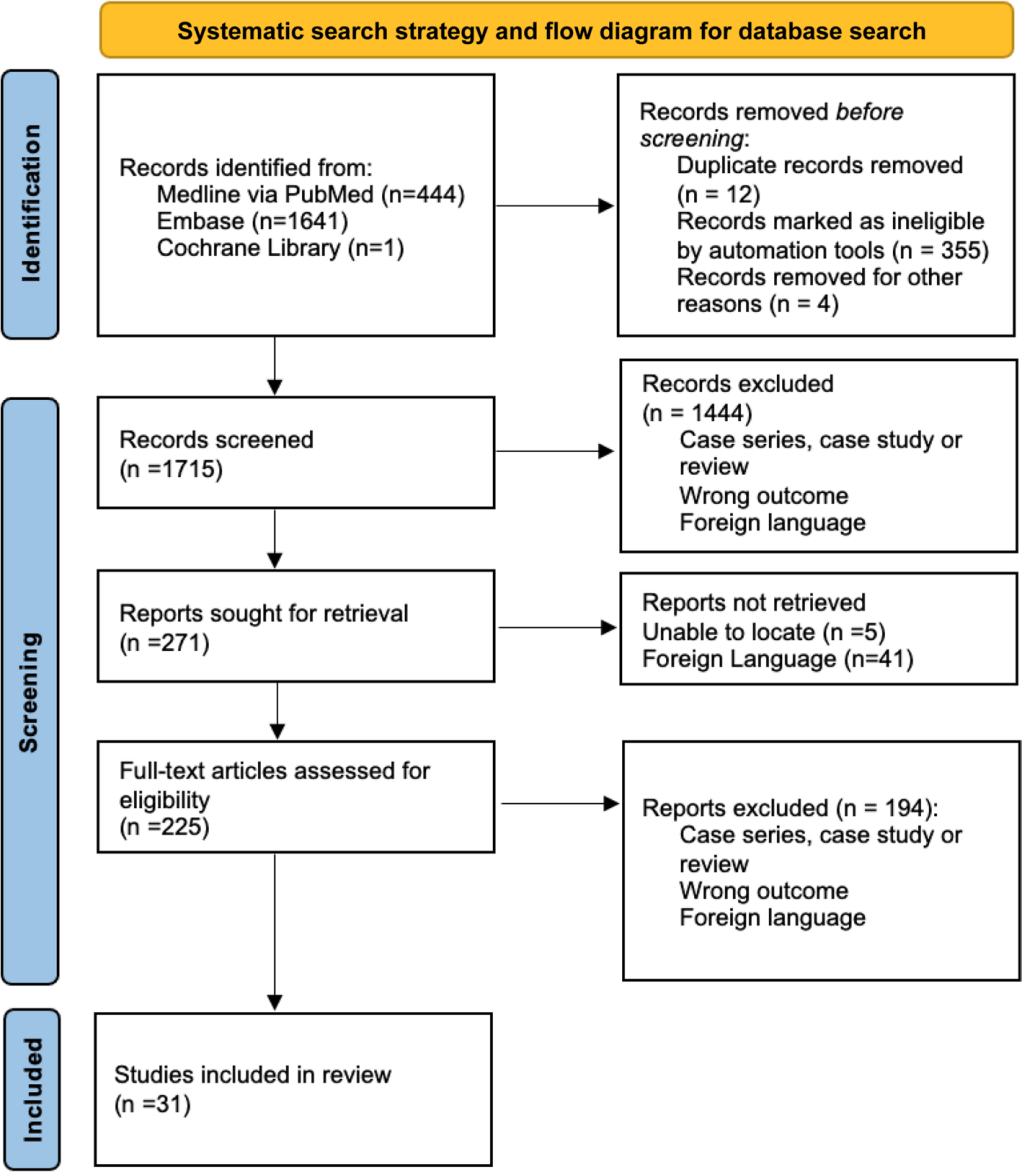
Identification of studies via databases and registers

A total of 1715 unique records were identified (Fig. 1). A total of 1444 records were excluded due to lack of relevance during title and abstract review. The remaining 225 articles underwent full-text review, yielding 31 articles for inclusion (outlined in Table 1).

**Table 1:** Summary Table of Systematic Review Findings

	Summary of Findings	Relevant References
Periocular Pigmentary Changes	Poliosis was commonly observed in patients with vitiligo, with reported prevalence of 5.9% [5], 16% [6], and 18% [7]. One study reported that of six patients with both uveitis and vitiligo, 67% also had poliosis [8]. Partial or total brow whitening was noted in 13.4% of vitiligo patients by another study [6]. Periocular depigmentation is another common feature of vitiligo. One study reported lid depigmentation in 48% of the vitiligo group [6] while other studies reported periocular involvement in 47-49% [9, 10]. Presence of periorbital depigmented patches was associated with a 58-fold increased risk for ocular findings [odds ratio (OR) 58.579, 95% CI 3.119–1100.3] [11]. Corneal fluorescein staining (CFS) positivity, reported as significantly higher in vitiligo patients compared to healthy controls, was also found to be more prominent in patients with periocular involvement but this difference was not statistically significant [10]. One study observed reductions in Schirmer test scores when comparing patients with periocular depigmentation to those without periocular depigmentation and to healthy controls. They found that patients with acrofacial vitiligo had significantly lower Schirmer test scores than patients with generalized vitiligo (*p* = 0.001) [12]. Another study found Schirmer test scores were lower in patients with periocular vitiligo compared to healthy controls, but this difference was not statistically significant [13]. Periocular vitiligo may also be related to measures of dry eye disease, including tear break up time (TBUT), Rose Bengal scores, and tear osmolarity [12–14]. Patients with acrofacial vitiligo had significantly lower TBUT (*p* = 0.001) and significantly higher Rose Bengal scores (*p* = 0.011) than patients with generalized vitiligo [12]. Compared to healthy controls, patients with periocular involvement also had significantly lower TBUT (*p* = 0.005) [13]. Moreover, 62% of periocular vitiligo patients compared to 28% of healthy controls had TBUT values less than 10 s, a statistically significant difference (*p* < 0.001) [13]. In terms of tear osmolarity, patients with vitiligo with ocular involvement had significantly higher tear osmolarity values than those without ocular involvement and healthy controls (*p* = 0.02, *p* < 0.001, respectively) [14].	Albert, D.M., J.J. Nordlund, & A.B. Lerner (1979) [6] Bulbul Baskan, E., et al. (2006) [11] Dertlioğlu, S.B., et al. (2016) [9] Dogan, A.S., et al. (2015) [10] Ayotunde, A. & G. Olakunle (2005) [5] Nordlund, J.J., et al. (1981) [8] Güngör, Ş., et al. (2015) [12] Serin, D., et al. (2012) [13] Erdur, S.K., et al. (2018) [14] Gopal, K.V.T., et al. (2007) [7]
Dry Eye Disease	In evaluating for dry eye disease in vitiligo patients, studies overwhelmingly reported that vitiligo patients had lower Schirmer scores compared to healthy controls, but at a difference that was not statistically significant [10, 12–15]. Only one study reported a statistically significant reduction in Schirmer scores [16] compared to healthy controls. Other indicators of dry eye disease were more conclusive, such as reports of significantly lower fluorescein breakup (FBU) scores [10], significantly higher Rose Bengal scores [12], and significantly lower tear breakup scores (TBUT) in patients with vitiligo [12–15]. One study reported that tear osmolarity was significantly higher among vitiligo patients compared to healthy controls [14]. Ocular surface disease index (OSDI) scores were higher in vitiligo patients compared to controls, either approaching significance (*p* = 0.071) [15] or achieving significance [10, 14], (*p* < 0.001 and *p* = 0.001, respectively). To better understand the potential mechanism of dry eye disease in vitiligo patients, one study used conjunctival impression cytology and observed that 31.7% of patients with periocular vitiligo compared to 8% of healthy controls had grade 2–3 changes per Nelson classification, a statistically significant difference [13]. Of the studies that investigated Meibomian gland dysfunction (MGD) [10, 15], one reported that there was no difference in MGD between vitiligo patients and healthy controls while the other reported a statistically significant difference. Meibomoscores in patients with periocular involvement did not differ significantly from those without periocular involvement [15].	Dogan, A.S., et al. (2015) [10] Güngör, Ş., et al. (2015) [12] Serin, D., et al. (2012) [13] Karadag, R., et al. (2016) [16] Erdur, S.K., et al. (2018) [14] Palamar, M., et al. (2017) [15]
Cataracts	Several studies reported an absence of notable slit lamp findings, observing clear ocular media with no corneal infiltrates [17] and no anterior lenticular opacities [17–19]. However, one study described three patients in their mid-thirties with posterior subcapsular cataract of no evident cause, [6] and another study described two patients with mild lens nuclear sclerosis in both eyes [19]. Karadag et al. reported a statistically significant difference in prevalence of punctate lenticular opacities in vitiligo patients compared to controls (30.4% and 10.5%, respectively) (*p* = 0.033)), [16] but it seems that overall, there was no significant difference in the incidence of cataracts in vitiligo patients compared to healthy controls (*p* = 0.6854) [20, 21].	Albert, D.M., J.J. Nordlund, & A.B. Lerner (1979) [6] Karadag, R., et al. (2016) [16] Fouad, Y.A., et al.(2020) [20] El-Mofty, A.M. and A. El-Mofty (1979) [17] Khurrum, H., K.M. AlGhamdi, & E. Osman (2016) [21] Mostafa W.Z., et al. (2015) [18] Gass, J.D. (1981) [19]
Iris Atrophy & Pigmentary Abnormalities	Several studies reported findings of iris atrophy and/or pigmentary abnormalities [6, 7, 16, 20, 22–25]. While findings of iris atrophy were overall higher in vitiligo patients compared to healthy controls, the prevalence was variable, ranging from 0%-25.5% [6, 23, 24]. In the study that identified focal iris atrophy in 25.5% of vitiligo patients, findings ranged from subtle transillumination defects to small atrophic patches [23]. One study reported racial differences among vitiligo patients, with white patients having higher prevalence of iris transillumination compared to black patients (23% and 5.5%, respectively) [22]. Iris hypopigmentation in vitiligo patients ranged from 5-23% compared to 1-7.1% in healthy controls [7, 23]. One study reported iritis in 2.68% of vitiligo patients compared to 0% of healthy controls. The iritis was associated with heterochromia [6]. Another study reported iritis in 12.5% of vitiligo patients [25]. There does not appear to be a significant difference in iris color between the vitiligo and control groups [7, 16].	
Elevated Intraocular Pressure & Glaucoma	Studies reported no significant difference in intraocular pressure (IOP) among vitiligo patients compared to healthy controls [5, 9, 16, 20]. One study diagnosed glaucoma in 2.2% of patients with vitiligo (2/90) compared to 0% of controls. The 2 patients who had glaucoma had generalized vitiligo [21]. One study did find a statistically significant difference in normal tension glaucoma (NTG) in vitiligo patients compared to healthy controls (18% and 0%, respectively) (*p* = 0.04). Two-thirds of the patients with NTG had periorbital lesions [9]. Rogosic et al. reported primary open angle glaucoma in 57% (24/42) of their vitiligo patients with the duration of vitiligo nearly two-fold longer in patients with both vitiligo and glaucoma than in those with vitiligo alone (*p* < 0.001) [26]. They identified a 4.4-fold risk of developing glaucoma in patients aged 56 and older compared to those younger than age 56. When corrected for age, they found a 92% probability of association between duration of vitiligo (greater than 13 years) and development of glaucoma [26]. The only study that investigated retinal nerve fiber layer (RNFL) thickness found that mean RNFL thickness outside the disc margin was significantly lower in the vitiligo group compared to controls (*p* = 0.02), and that mean RNFL thickness beneath the measuring ellipse in the superior sector of both eyes were significantly lower than controls [27]. The significance of these findings is unclear.	Dertlioğlu, S.B., et al. (2016) [9] Ayotunde, A. & G. Olakunle (2005) [5] Karadag, R., et al. (2016) [16] Fouad, Y.A., et al.(2020) [20] Khurrum, H., K.M. AlGhamdi, & E. Osman (2016) [21] Ornek, N., et al. (2013) [27] Rogosić, V., et al. (2010) [26]
Uveitis	Studies found little evidence of increased rates of uveitis among those with vitiligo, as illustrated by no significant difference in prevalence of ocular inflammation between vitiligo and control groups [21–24]. A single study did report a slightly increased prevalence of active uveitis– either iridocyclitis, chorioretinitis, or both – among those with vitiligo (9/112 patients with vitiligo vs. 0/372 healthy controls) [6].	Cowan, C.L., Jr., et al.(1986) [22] Khurrum, H., K.M. AlGhamdi, & E. Osman (2016) [21] Biswas, G., et al. (2003) [23] Fleissig, E., et al. (2018) [24]
Retina & Choroid	Several studies reported normal fundal examinations in patients with vitiligo [17, 18, 21, 28]. Nevertheless, observed choroid pigment abnormalities in vitiligo patients included prominent choroidal pattern (36%) [6] and tigroid retina (9.8%) [16]. Prevalence of choroidal nevi varied greatly in the reviewed studies, ranging from 0-32.2% [20, 22, 24]. One study reported that there was a significantly higher prevalence of choroidal nevi in white compared to Black patients [22]. One study reported statistically reduced subfoveal choroidal thickness (*p* = 0.0002) [20] while another, which utilized OCT imaging, found no statistically significant difference in outer nuclear layer thickness, total macular thickness, or thickness of the macular RNFL, ganglion cell layer, inner nuclear layer, inner plexiform player, or outer plexiform layer between vitiligo patients and controls [29]. Several studies showed evidence of higher retinal pigmentary abnormalities in the vitiligo groups, including focal retinal pigment epithelium (RPE) hyperpigmentation, focal RPE hypopigmentation/atrophy, RPE mottling, and/or chorioretinal degeneration/scarring [20, 24, 30, 31]. Of the studies that reported on patients’ visual acuity, most [5, 16, 17, 29] reported normal visual acuity or no significant difference in visual acuity in vitiligo patients compared to healthy controls. Only one study reported significantly lower corrected visual acuity in vitiligo patients compared to controls (*p* = 0.0109) but this study was limited by a small sample size [20]. Another study reported that 95% of vitiligo patients (107/112 patients) had a visual acuity of 20/30 or better, and the remaining 5 patients’ impaired visual acuity was attributable to chorioretinitis-like lesions in 3 patients and other disease processes [6]. Similarly, perimetry measurements were reported as normal or not significantly different in vitiligo patients compared to controls [5, 16, 17]. The results of studies utilizing electroretinography (ERG) in vitiligo patients were varied. While some studies reported normal ERG results with no significant change in wave amplitudes and wave implicit times [18, 32], others reported ERG findings suggestive of impaired retinal electrophysiological function in vitiligo patients [19, 29, 33]. Shoeibi et al. concluded that the mean rod response b-wave, standard combined a- and b-waves, single-flash cone response b-wave and the 30-Hz flicker (N1-P1) amplitudes were significantly lower in vitiligo patients compared to age-matched healthy controls [33]. Similarly, Aydin et al. found that the mean mfERG-c P1 and mfERG-p P1 amplitudes were significantly lower in the vitiligo group compared with controls (*p* = 0.002 and *p* = 0.006, respectively) [29]. One group identified moderately to severely abnormal rod and cone function in both eyes of all ten vitiligo patients who were tested [19].	Albert, D.M., J.J. Nordlund, & A.B. Lerner (1979) [6] Ayotunde, A. & G. Olakunle (2005) [5] Karadag, R., et al. (2016) [16] Cowan, C.L., Jr., et al.(1986) [22] Fouad, Y.A., et al.(2020) [20] Aydin, R., et al. (2018) [29] El-Mofty, A.M. and A. El-Mofty (1979) [17] Khurrum, H., K.M. AlGhamdi, & E. Osman (2016) [21] Biswas, G., et al. (2003) [23] Fleissig, E., et al. (2018) [24] Wagoner, M.D., et al. (1983) [30] Shoeibi, N., et al. (2014) [33] Gopal, K.V.T., et al. (2007) [7] Lerner, A.B., J.J Nordlund, & D.M Albert (1977) [25] Mehran, G., et al. (2014) [34] Mostafa W.Z., et al. (2015) [18] Perossini, M., et al. (2010) [28] Shoeibi, N., et al. (2016) [32] Gass, J.D. (1981) [19]

### I. Periocular pigmentary changes

Periocular skin depigmentation is a common feature of vitiligo. Among studies with documented rates of periocular depigmentation, most reported a prevalence between 40–49% [6, 9–11, 15]. Additionally, the presence of poliosis (or whitening) of the eyelashes and eyebrows is a well-established finding in vitiligo, albeit less common than periocular depigmentation [5, 6, 8]. The relationship between these periocular pigmentary abnormalities and the presence of ocular pathology is not as readily apparent.

A number of studies [10–15] found that those with periocular skin depigmentation are at higher risk for ocular abnormalities than those without, including peripapillary atrophy, retinal and iris pigmentary abnormalities, and tear film dysfunction (as measured by Schirmer’s test, tear film break-up time, and tear osmolarity). One study [11] of 45 patients with vitiligo cites a 58-fold increase risk for ocular findings in patients with periocular vitiligo compared to those without periocular involvement, suggesting localization of lesions might impact pathophysiology. However, the size of the study and the narrow range of ocular findings precluded any definitive conclusions. Albert et al. [6] reported eyelid depigmentation in 48.2% and poliosis in 16.1% of subjects with vitiligo versus 0% in the control group. Of the subjects with eyelid depigmentation, 40.7% were also affected by discrete atrophic areas in the fundus, iris atrophy, or iritis.

Many included studies recorded the distribution or vitiligo subtype of their subjects, but did not provide details to evaluate for an association between vitiligo subtype and presence or severity of ocular findings. Gungor et al. compared patients with acrofacial to generalized vitiligo and found significantly lower Schirmer test scores and TBUT values and significantly higher Rose Bengal scores in patients with acrofacial vitiligo [12]. Considering the subtype of vitiligo is an important element for future research as ocular manifestations seen in vitiligo subtypes that affect periocular skin may be able to provide insight into disease pathogenesis.

### II. Dry Eye Disease (DED)

This SR revealed substantial evidence linking vitiligo to clinical evidence of dry eyes and ocular surface disease. Compared to healthy controls, patients with vitiligo were found to have significantly faster tear break-up times [10, 12–14], significantly higher tear osmolarity, [14] and significantly worse corneal surface staining (measured with either fluorescein or Rose Bengal stains) [10, 15]. One study [13] found that 31.7% of patients with periocular vitiligo versus 8% of controls met grade 2–3 classification using Nelson’s classification for squamous metaplasia of the conjunctiva. This points to an association between periocular vitiligo and DED, potentially mediated by a shared, underlying inflammatory etiology that adversely affects ocular surface goblet and epithelial cells.

The results of Schirmer’s tests were less conclusive, as only one study [16] found a significantly decreased Schirmer’s in vitiligo patients compared to healthy controls. However, there was a trend (approaching significance) toward decreased Schirmer’s tests in vitiligo patients in every other study reporting Schirmer’s findings, [10, 12–15] suggesting that patients with vitiligo may have reduced tear production. Subjectively, patients with vitiligo had significantly [10, 14, 29] or near-significantly [15] higher scores on the Ocular Surface Disease Index (OSDI), a 12-item questionnaire evaluating a patient’s symptoms and their effect on quality of life [35].

Of the studies that evaluated for meibomian gland dysfunction, one [15] identified a significant difference between meiboscores (upper and total eyelids) between patients with vitiligo and healthy controls while the other found no significant difference in meibomian gland function [10]. There was no significant difference in meiboscores in patients with periocular involvement versus those without [15].

### III. Cataracts

Amongst studies reporting lenticular exams, no significant difference in the prevalence of cataracts was detected between those with vitiligo and healthy controls [17, 20, 21]. A single study [16] did find a higher rate of punctate lenticular opacities in those with vitiligo but did not see any increase in visually significant lenticular opacities. Another study anecdotally describes three patients with vitiligo in their mid-30’s who developed posterior subcapsular cataracts [6].

### IV. Iris atrophy

Given the pigmented nature of the iris (specifically the iris pigment epithelium), several studies examined the relative presence of iris atrophy and/or pigmentary abnormalities (such as heterochromia and hypopigmentation) in vitiligo [6, 16, 20, 22–24]. Half of these found significantly higher rates of iris atrophy (i.e. transillumination defects) or iris hypopigmentation in patients with vitiligo compared to healthy controls [6, 20, 23]. One study [22] which compared rates of iris atrophy between different racial groups with vitiligo, observed more iris atrophy in white versus black patients, though both groups displayed some degree of iris atrophy. Karadag et al. noted no difference in iris color between vitiligo and control groups [16] while Albert et al. reported lighter iris color in 2 of 3 patients with active iritis [6]. Overall, the balance of observations seems to suggest higher rates of iris atrophy or hypopigmentation in those with vitiligo, albeit without any reported functional consequence.

### V. Elevated intraocular pressure and glaucoma

No studies found a statistically significant difference in intraocular pressure (IOP) between patients with vitiligo and healthy controls [16, 20, 36]. Similarly, the single study to quantify retinal nerve fiber layer (RNFL) thickness found a few small, non-specific differences between those with and without vitiligo, but ultimately concluded that RNFL thickness does not seem to be affected by the disease [27].

Nevertheless, one group compared 49 patients with vitiligo to 20 healthy controls and identified a rate of 18.4% of normal-tension glaucoma (NTG) among those with vitiligo versus 0% in the control group [9]. The mean age of the patients with NTG was 26.22 years, and was not significantly different from those patients without NTG. Interestingly, of the patients with NTG, two-thirds had periorbital lesions [9].

Rogosić et al. found an extremely high prevalence of primary open angle glaucoma (POAG) (24/42; 57%) among patients with vitiligo referred for eye exams [26]. There was no significant difference in terms of age or sex in those who did and did not have POAG, but they did conclude that the duration since vitiligo onset was nearly twofold longer in patients with both vitiligo and POAG than in those with vitiligo alone. Intraocular pressures reported were within the normal range and no ongoing treatment for glaucoma was specified. After correcting for age, the probability of association between risk of glaucoma and duration of vitiligo was determined to be 92% (with a majority of patients older than 56 years having glaucoma and a minority in patients younger than 56 years) [26].

Khurrum et al. studied 90 patients with periocular and/or facial vitiligo with 90 age-matched healthy controls and detected no difference in glaucoma prevalence between those with and without vitiligo. Both patients who developed glaucoma used long-term periorbital topical corticosteroids, but the majority of patients who used long-term topical corticosteroids on the eyelids/periorbital region did not develop glaucoma (17/19; 89.5%) [21]. However, the average age of patients with vitiligo in this study was 32.54 years, much lower than the average age of glaucoma onset observed in the general population.

### VI. Uveitis

Perhaps because of the symptomatology of Vogt-Koyanagi-Harada and Alezzandrini syndrome, many of the included studies investigated the possible link between vitiligo and uveitis. Overall, the studies found little evidence of increased rates of uveitis among those with non-syndromic vitiligo, as illustrated by no significant difference in prevalence of ocular inflammation between vitiligo and control groups [21–24]. A single study did report a slightly increased prevalence of active uveitis – either iridocyclitis, chorioretinitis, or both – among those with vitiligo (9/112 patients with vitiligo vs. 0/372 healthy controls), positing that a spectrum of vitiligo-associated ocular inflammatory conditions may exist, with VKH lying on the extreme end of the spectrum [6].

Two studies investigated the prevalence of vitiligo in patients with idiopathic uveitis, observing that vitiliginous lesions are more common in those presenting to ophthalmology clinic with symptomatic uveitis than in the general population [8, 30]. Clearly, stating that people with uveitis are more likely to have vitiligo is not equivalent to claiming that those with vitiligo are more likely to have uveitis. Nonetheless, it does speak to a possible underlying association between the two inflammatory conditions.

### VII. Retina and choroid

Substantial evidence linking vitiligo to changes in the retina and choroid exists. Two studies found a higher rate of prominent choroidal pattern (aka “tigroid fundus”) in vitiligo compared to healthy controls [6, 16]. Similarly, others found a higher prevalence of retinal pigmentary abnormalities – focal RPE hyperpigmentation, focal RPE hypopigmentation/atrophy, RPE mottling, and/or chorioretinal degeneration/scarring – in patients with vitiligo than in patients without, [6, 20, 22, 24, 30, 31] with reported percentages ranging from 5 to 40%. Two studies also noted the relatively common appearance of choroidal nevi in those with vitiligo, [22, 24] though another that compared these patients with healthy controls found no difference in the frequency of choroidal nevi between the two groups [20]. The single study to evaluate subfoveal choroidal thickness did find a significantly thinner choroid in patients with vitiligo [20]. In contrast, a different analysis discovered no differences in multiple optical coherence tomography (OCT) parameters (including total macular thickness, RNFL thickness, ganglion cell layer thickness, and presence of healthy ellipsoid zone) in patients with vitiligo than in otherwise healthy controls [29].

The trend of evidence strongly suggests that there is increased retinal and choroidal pigmentary abnormalities in vitiligo. But, as most of these pigmentary changes and areas of atrophy are peripheral, it remains unclear if there is any associated functional consequence. As previously mentioned, there does not appear to be any damage to the ellipsoid zone or atrophy of the central macula on OCT [29]. However, electroretinograms (ERGs) in vitiligo suggest the possibility of some functional retinal impairment. One study revealed significantly lower mean mfERG P1 amplitudes compared to controls, [29] while another demonstrated decreased mean rod response b-wave, standard combined a- and b-waves, and 30-Hz flicker amplitudes when contrasted with age-matched norms [33].

Nonetheless, patients [29] with decreased mfERG P1 amplitudes maintained best-corrected visual acuity (BCVA) of 20/20, indicating little visual impairment. The other study reporting ERG results did not include BCVA in their results. Other studies included in this SR, however, did include BCVA, and the majority [16, 17, 29, 36] showed either normal BCVA or BCVA no different than control groups. One study [6] of 112 patients with vitiligo demonstrated only 5 patients with BCVA worse than 20/30, and all 5 were attributable to other disease processes. Additionally, the perimetry evaluations (in the absence of glaucoma) showed either normal visual fields [17] or visual fields equivalent to healthy controls [16]. One study did find slightly worse vision in vitiligo patients compared to controls, but the small sample size and possible presence of confounding variables indicated the need for further assessment [20].

## Discussion

Aside from the well-established connection between the vitiliginous pigmentary changes and ocular inflammation that can coexist in Vogt-Koyanagi-Harada (VKH) and Alezzandrini syndromes, the link between non-syndromic vitiligo and ocular inflammation is not well understood.

Vitiligo can affect skin, hair, and mucous membranes anywhere on the body, but the most common subtypes—generalized and acrofacial vitiligo—share a predilection for the face. Thus, patients with vitiligo experience periocular pigmentary changes and poliosis more often than the general population. There is some level of evidence – though far from definitive – indicating that those with periocular depigmentation are at greater risk of certain structural ocular abnormalities than the vitiligo population in general. It would be prudent to pay close attention to symptoms of dry eyes and intraocular pigmentary changes in patients with periocular vitiligo.

Overall, the strongest link between vitiligo and ophthalmic pathology seems to lie with tear film abnormalities and ocular surface disease. Though DED is a multifactorial and a common entity in the general population, it is known to have an underlying inflammatory component, suggesting a possible etiologic link with the inflammatory nature of vitiligo [37]. Additionally, compared to the general US population, patients with vitiligo have a statistically significant higher prevalence of Sjögren syndrome, which may contribute to the high rates of DED identified in this SR [38]. The evidence for DED strongly establishes evidence for a *functional* consequence of structural abnormalities, with the OSDI results indicating symptomatically significant pathology [35].

Scant evidence exists associating vitiligo to the presence of cataracts. A single study identified three patients with vitiligo in their mid-30’s who developed posterior subcapsular cataracts. However, the authors posit that these were most likely secondary to a remote inflammatory event [6]. Nevertheless, patients with vitiligo can be treated with intermittent courses of systemic corticosteroids, a confounding factor known to contribute to cataract formation. Further research is needed to determine whether patients who received course(s) of systemic steroids have higher risk of cataract formation versus those who did not receive systemic steroids.

The prevalence of glaucoma in individuals under age 55 is rare (< 1%). This SR did identify a few studies that suggest a significantly higher rate of glaucoma in patients with vitiligo, specifically the normal-tension form of glaucoma, which is not associated with elevated intraocular pressures. The rates of glaucoma were significantly higher in patients older than 55 years and those with greater duration of time since diagnosis of vitiligo [9]. Amongst studies noting a higher rate of glaucoma among the vitiligo population, there is no mention of the level of glaucoma severity, level of functional impairment, or what treatments had been instituted. Additionally, several studies did not report the parameters used to diagnose glaucoma. Nevertheless, it is important to inquire about known risk factors, namely family history and steroid use (including formulation, dose, and duration) and carefully examine the optic nerve of any patient with vitiligo undergoing an ophthalmic examination. Given our current knowledge, older patients (> age 55 years) with vitiligo who took repeated courses of systemic steroids or used periorbital topical steroids should likely be evaluated for glaucoma.

The relationship between vitiligo and uveitis is disputed and is based on the current understanding of VKH symptomatology. Some hypothesize that VKH lies at the extreme end of the ocular inflammatory spectrum, with ocular manifestations of non-syndromic vitiligo lying at a less extreme end. VKH is characterized pathologically by diffuse thickening within the uveal tract secondary to non-necrotizing granulomatous inflammation, with inflammatory cells found in close proximity to melanocytes. Vitiliginous skin is identified in less than one-quarter of patients with VKH [39]. Overall, those with vitiligo without symptomatic ocular pathology have very low prevalence of chronic or acute uveitis, in line with rates found in healthy controls. In patients with idiopathic ocular inflammation, however, attention should be paid to the presence of depigmented or vitiliginous skin lesions.

Despite some evidence of abnormal ERG responses, data regarding good visual acuity and perimetry suggests that the retinal and choroidal findings have limited to no impact on visual function. Histopathologic changes include degeneration of photoreceptor cells and disruption of the outer segment/ RPE interdigitation similar to RP, a finding that has also been identified in vitiligo mutant mice. Tang et al. reported abnormal flash ERG findings, including delayed a- and b-waves, that correlated to histopathologic abnormalities such as short and disoriented rod outer segments leading to retinal separation from the pigment epithelium [40]. In a Mitf mouse model of vitiligo, Bora et al provided evidence of naturally occurring retinal detachment in mutant mice [41].

Limitations relevant to this study include the review of articles exclusively in the English language. Additionally, techniques used in various manuscripts can be particularly user-dependent (i.e., ERG) and therefore results from these studies should be interpreted with caution.

## Conclusion

In summary, it appears that patients with vitiligo are at higher risk for DED and for ocular pigmentary abnormalities. However, outside of the potential impact of dry eyes, it does not seem likely that there are any functional consequences of these ocular manifestations (e.g. impaired visual acuity or visual fields).

## Data Availability

The datasets used and/or analyzed during the current study available from the corresponding author on reasonable request.

## Abbreviations

SR: Systematic review
OSDI: Ocular Surface Disease Index
IOP: Intraocular pressure
RNFL: Retinal nerve fiber layer
POAG: Primary open angle glaucoma
RPE: Retinal pigmente epithelium
OCT: Optical coherence tomography
ERG: Electroretinogram
BCVA: Best-corrected visual acuity
VKH: Vogt-Koyanagi-Harada
DED: Dry eye disease
